# BrainIAK: The Brain Imaging Analysis Kit

**DOI:** 10.52294/31bb5b68-2184-411b-8c00-a1dacb61e1da

**Published:** 2022-02-16

**Authors:** Manoj Kumar, Michael J. Anderson, James W. Antony, Christopher Baldassano, Paula P. Brooks, Ming Bo Cai, Po-Hsuan Cameron Chen, Cameron T. Ellis, Gregory Henselman-Petrusek, David Huberdeau, J. Benjamin Hutchinson, Y. Peeta Li, Qihong Lu, Jeremy R. Manning, Anne C. Mennen, Samuel A. Nastase, Hugo Richard, Anna C. Schapiro, Nicolas W. Schuck, Michael Shvartsman, Narayanan Sundaram, Daniel Suo, Javier S. Turek, David Turner, Vy A. Vo, Grant Wallace, Yida Wang, Jamal A. Williams, Hejia Zhang, Xia Zhu, Mihai Capotă, Jonathan D. Cohen, Uri Hasson, Kai Li, Peter J. Ramadge, Nicholas B. Turk-Browne, Theodore L. Willke, Kenneth A. Norman

**Affiliations:** a Princeton Neuroscience Institute, Princeton University, Princeton, NJ; b Work done while at Parallel Computing Lab, Intel Corporation, Santa Clara, CA; c Department of Psychology, Columbia University, NY, NY; d International Research Center for Neurointelligence (WPI-IRCN), UTIAS, The University of Tokyo, Japan; e Work done while at Princeton Neuroscience Institute, Princeton University, Princeton, NJ; f Department of Psychology, Yale University, New Haven, CT; g Department of Psychology, University of Oregon, Eugene, OR; h Department of Psychology, Princeton University, Princeton, NJ; i Department of Psychological and Brain Sciences, Dartmouth College, Hanover, NH; j Parietal Team, Inria, Neurospin, CEA, Université Paris-Saclay, France; k Department of Psychology, University of Pennsylvania, Philadelphia, PA; l Max Planck Research Group NeuroCode, Max Planck Institute for Human Development, Berlin, Germany; m Max Planck UCL Centre for Computational Psychiatry and Ageing Research, Berlin, Germany; n Department of Computer Science, Princeton University, Princeton, NJ; o Brain-Inspired Computing Lab, Intel Corporation, Hillsboro, OR; p Department of Electrical Engineering, and the Center for Statistics and Machine Learning, Princeton University, Princeton, NJ

**Keywords:** MVPA, fMRI analysis, high-performance computing, machine learning, fMRI simulator, tutorials

## Abstract

Functional magnetic resonance imaging (fMRI) offers a rich source of data for studying the neural basis of cognition. Here, we describe the Brain Imaging Analysis Kit (BrainIAK), an open-source, free Python package that provides computationally optimized solutions to key problems in advanced fMRI analysis. A variety of techniques are presently included in BrainIAK: intersubject correlation (ISC) and intersubject functional connectivity (ISFC), functional alignment via the shared response model (SRM), full correlation matrix analysis (FCMA), a Bayesian version of representational similarity analysis (BRSA), event segmentation using hidden Markov models, topographic factor analysis (TFA), inverted encoding models (IEMs), an fMRI data simulator that uses noise characteristics from real data (fmrisim), and some emerging methods. These techniques have been optimized to leverage the efficiencies of high-performance compute (HPC) clusters, and the same code can be se amlessly transferred from a laptop to a cluster. For each of the aforementioned techniques, we describe the data analysis problem that the technique is meant to solve and how it solves that problem; we also include an example Jupyter notebook for each technique and an annotated bibliography of papers that have used and/or described that technique. In addition to the sections describing various analysis techniques in BrainIAK, we have included sections describing the future applications of BrainIAK to real-time fMRI, tutorials that we have developed and shared online to facilitate learning the techniques in BrainIAK, computational innovations in BrainIAK, and how to contribute to BrainIAK. We hope that this manuscript helps readers to understand how BrainIAK might be useful in their research.

## INTRODUCTION

Cognitive neuroscientists have come a long way in using functional magnetic resonance imaging (fMRI) to help answer questions about cognitive processing in the brain. A variety of methods have been developed, ranging from univariate techniques to multivariate pattern analysis (MVPA) methods [1–4]. A large number of toolboxes are now available that implement these pattern analysis methods, including, for example the Princeton MVPA Toolbox [5], the Decoding Toolbox [6], CoSMoMVPA [7], Nilearn [8], and PyMVPA [9] (for a full list see https://github.com/ohbm/hackathon2019/blob/master/Tutorial_Resources.md). Scientists can choose which toolbox to use based on the analysis that they wish to perform and the programming language they wish to use.

In this work, we describe the Brain Imaging Analysis Kit (BrainIAK (RRID:SCR 014824), https://brainiak.org), an open-source Python package that implements computationally optimized solutions to key problems in advanced fMRI data analysis, focusing on analysis steps that take place after data have been preprocessed and put in matrix form. BrainIAK can be viewed as a “Swiss army knife” for advanced fMRI analysis, where we are constantly striving to add new tools. Presently, BrainIAK includes methods for running intersubject correlation (ISC) [10] and intersubject functional correlation (ISFC) [11, 12], functional alignment via the shared response model (SRM) [13], Bayesian Representational Similarity Analysis (RSA) [14, 15], event segmentation [16], dimensionality reduction via topographic factor analysis (TFA) [17], and inverted encoding models (IEMs) [18, 19].

To avoid duplication across packages, BrainIAK leverages available methods in other packages – it is well integrated with Nilearn (https://nilearn.github.io/index.html) [20] and extensively uses scikit-learn (https://scikit-learn.org/) [21] for machine learning algorithms. The functions in BrainIAK are optimized to run on high-performance compute (HPC) clusters for efficient execution on large datasets. The same code can be executed on a laptop or an HPC cluster, saving significant time in refactoring the code to run in an HPC environment. BrainIAK also includes a detailed set of tutorials [22] that are didactic in nature; the tutorials include very detailed steps and helper functions that facilitate learning and implementing some of the methods, including materials relevant to running on HPC clusters. Scientists can also use BrainIAK’s simulator [23] to create model-based patterns of activity at the voxel level, without going through the expensive and time-consuming process of data collection. The package is released with an open-source license and is free to use on a variety of platforms. The BrainIAK package welcomes contributions from the community, and new methods are continuously added to the package.

## METHODS IN BRAINIAK

In the following sections, we present an overview of each of the methods presently included in BrainIAK and an accompanying example notebook. For each method, we list the data analysis problem that it is meant to solve and how it solves that problem. The notebooks also contain an annotated bibliography for each method, listing papers that have described and/or used this method. These example notebooks are not as didactic as the tutorials. Instead, the notebooks we provide here are integrated with the BrainIAK documentation, provide an overview of the technique, and allow users to quickly access code snippets for the method. Also, the notebooks include methods that are not covered in the tutorials such as Bayesian RSA [14, 15], TFA [17], IEMs [18, 19], BrainIAK’s simulator [23], and matrix-normal models [24]. All example notebooks are available at https://github.com/brainiak/brainiak-aperture, along with instructions on how to run them.

### Intersubject Correlation

#### The Problem: Measuring the Brain’s Response to Naturalistic Stimuli

One of the traditional goals of fMRI research is to measure the brain’s response to a particular stimulus, task, or other experimental manipulation. Typically, this approach relies on tightly controlled experimental designs – by contrasting two stimuli or tasks, or parametrically varying a particular experimental variable, we can isolate brain responses to the variable of interest. Experimentally isolating particular variables can reduce ecological validity; in response to this, cognitive neuroscientists have begun to adopt more naturalistic paradigms [25–30]. However, using naturalistic stimuli comes with its own set of challenges – in particular, if the stimuli are too complex to be modeled using a small set of regressors, the standard approach of relating a design matrix to the fMRI signal may not be practical.

#### The Solution

**Fig. 1.**
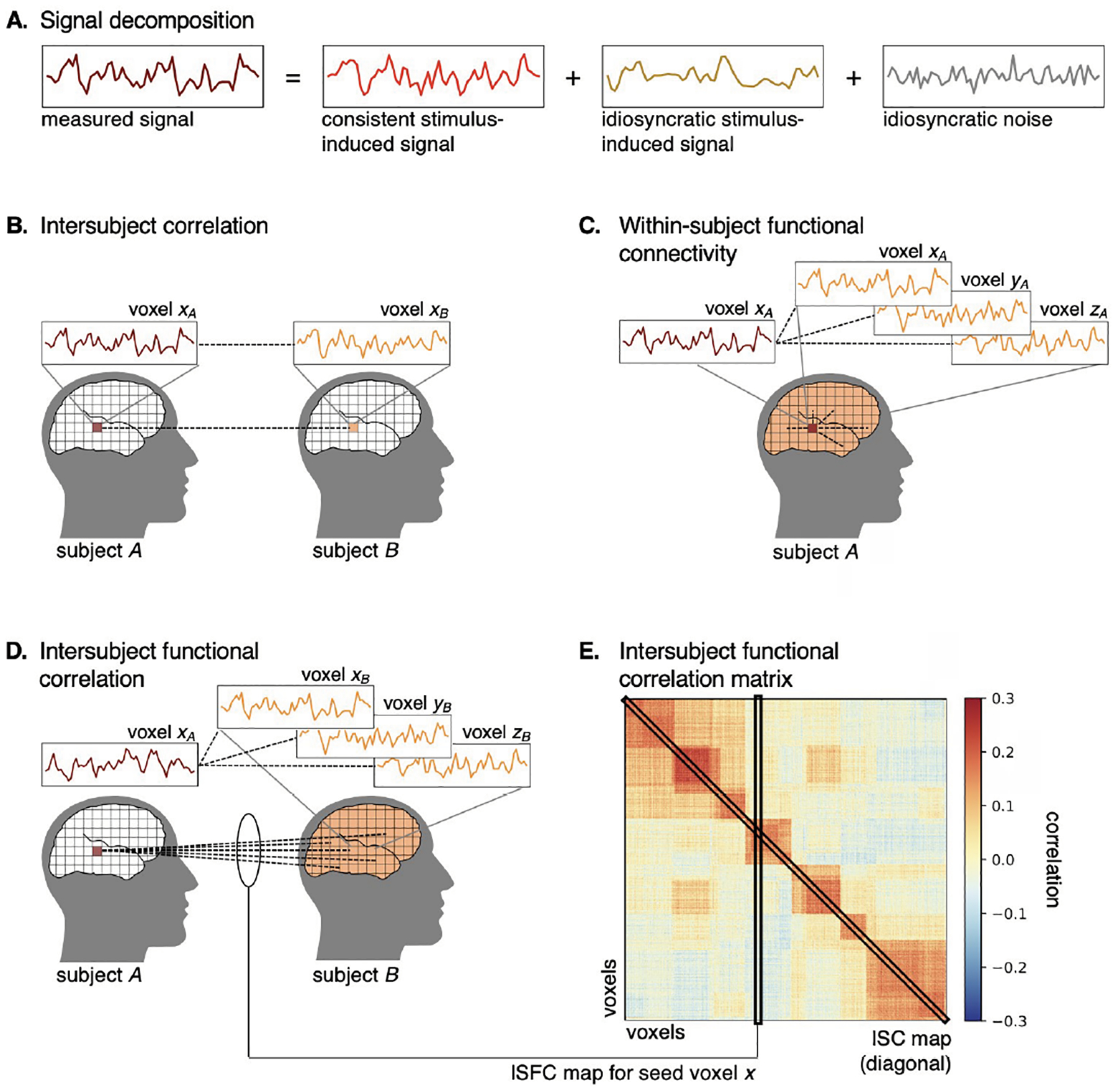
Schematic of ISC and ISFC analysis. A. The measured response time series (maroon) can be decomposed into three components: a consistent stimulus-induced component that is shared across subjects (red), an idiosyncratic stimulus-induced component (gold), and an idiosyncratic noise component (gray). B. ISC is computed between two homologous brain areas (maroon and orange) across subjects, thus isolating the shared, stimulus-induced signal from idiosyncratic signals. C. Typical functional connectivity analysis is computed within subjects across brain areas. D. ISFC is computed across both subjects and brain areas. ISFC analysis provides functional network estimation analogous to within-subject functional connectivity analysis, but isolates the shared, stimulus-induced signal and is robust to idiosyncratic noise. E. The diagonal of the ISFC matrix comprises the ISC values.

ISC analysis takes a different approach to this problem – instead of trying to fully describe the stimulus in a design matrix, ISC measures stimulus-evoked responses to naturalistic stimuli by isolating brain activity shared across subjects receiving the same stimulus [10, 12]. When experimental participants are presented with a stimulus such as a movie or a spoken story, their brain activity can be conceptually decomposed into at least two components: (1) a stimulus-related component that is synchronized across subjects due to the use of a common stimulus; and (2) a subject-specific component capturing both idiosyncratic stimulus-related signals (e.g., unique memory and interpretation) and nonstimulus-related signals (e.g., physiological noise; Figure 1A). ISC analysis measures the former (shared, stimulus-related) component, filtering out the latter (idiosyncratic) component (Figure 1B).

This shared signal can be driven by different features of the stimulus in different brain regions. For example, when listening to a spoken story, ISC in early auditory areas may be driven by acoustic features of the stimulus, whereas ISC in the association cortex may be driven by higher-level linguistic features of the stimulus. In this sense, ISC is agnostic to the content of the stimulus and serves as a measure of reliability of stimulus-evoked responses across subjects (or as a “noise ceiling” for model-based prediction across subjects [12, 31, 32]). This is particularly useful for complex, naturalistic stimuli where exhaustively modeling stimulus features may be difficult. This also allows us to leverage naturalistic stimuli to ask novel questions about brain organization. For example, high ISCs extend from early auditory areas to high-level association cortices during story-listening. However, if we temporally scramble elements of the story stimulus, this disrupts the narrative content of the story; in this case, we still observe high ISC in early auditory areas, but less so in higher-level cortices, suggesting that certain association areas encode temporally evolving narrative content [33, 34].

Several variations on ISC have been developed at both the implementational and conceptual levels. For example, ISCs may be computed in either a pairwise or leave-one-out fashion, both of which have associated statistical tests [12, 35, 36]. An important conceptual advance has been to compute ISC across brain areas using ISFC analysis [11, Figure 1D]. ISFC analysis allows us to estimate functional connectivity (FC) networks analogous to traditional within-subject FC analysis (Figure 1C). However, unlike traditional within-subject FC analysis, ISFC analysis isolates stimulus-driven connectivity and is robust to idiosyncratic noise due to head motion and physiological fluctuations [37]. Both ISC and ISFC can be computed using a sliding window to measure coarse fluctuations in the shared signal over time. Finally, rather than computing ISC on response time series, we can also apply the logic of ISC to multivoxel pattern analysis [1]. Intersubject pattern correlation analysis captures spatially distributed shared response patterns across subjects at each time point (e.g., [38]). Computing spatial ISC between all time points (the spatial analogue of ISFC) enables us to discover whether certain spatial response patterns are consistent or reemerge over time [16].

#### The Notebook

The accompanying notebook applies ISC analysis to an example fMRI story-listening dataset from the “Narratives” data collection [39, 40]. To reduce computational demands, we compute ISC on a time series averaged within each parcel extracted from a functional cortical parcellation [41]. We first demonstrate high ISC values extending from low-level auditory cortex to higher-level cortical areas during story listening. However, when listening to a temporally scrambled version of the stimulus, ISC is dramatically reduced in higher-level cortex areas, suggesting that these areas encode temporally evolving features of the stimulus (e.g., narrative context). We next perform a similar comparison between intact and scrambled story stimuli using traditional within-subject FC and ISFC analysis. The networks estimated using within-subject FC are similar across the two types of stimuli, while ISFC analysis yields very different networks for the intact and scrambled stories. BrainIAK also offers several nonparametric statistical tests for ISC and ISFC analysis, some of which are discussed in the notebook.

#### Compute Recommendations

The computational demands of ISC/ISFC analyses scale with the number of subjects, voxels, and timepoints (TRs); however, the memory demands of pairwise ISC analysis will increase more precipitously with the number of subjects. A small-scale (e.g., parcellation-based) ISC analysis with 30 subjects, 1,000 parcels, and a 300-TR duration runs in a couple of seconds on a typical personal computer. On the other hand, whole-brain voxelwise ISC analysis with 50,000 voxels may require 10 or more minutes to run and require several GB of memory. For large-scale ISC analyses, we recommend running the analysis on a distributed computing cluster. Basic ISC/ISFC analysis (as implemented in BrainIAK) requires a single process to operate on data across all subjects. However, some additional preprocessing can allow for parallelization across subjects. For example, in the leave-one-out approach, precomputing the average time series excluding each subject can allow the ISC computation to proceed in parallel; in the pairwise approach, ISC for each pair of subjects can be computed in parallel and then recombined. Note that ISC analysis proceeds independently for each brain variable (e.g., voxel or parcel), so ISC analysis can also be parallelized across voxels; for example, a whole-brain voxelwise ISC analysis with 50,000 voxels can be divided into 50 parallel jobs each running ISC analysis on a subset of 1,000 voxels.

ISFC analysis computes the correlation between all pairs of parcels or networks, and therefore, computational demand increases primarily with the number of voxels. Similar to ISC analysis, smaller-scale analyses (e.g., 30 subjects, 1,000 parcels, and 300 TRs) are easily computed on a personal computer, whereas whole-brain voxelwise analyses may require a computing cluster.

### Shared Response Model

#### The Problem: Aligning Brain Data across Participants

One of the main obstacles in leveraging brain activity across subjects is the considerable heterogeneity of functional topographies from individual to individual. Variability in functional–anatomical correspondence across individuals means that even high-performing anatomical alignment does not ensure fine-grained functional alignment (e.g., [42]). As an example, multivoxel pattern analysis models that perform well within subjects often degrade in performance when evaluated across subjects (e.g., [43, 44]).

#### The Solution

**Fig. 2.**
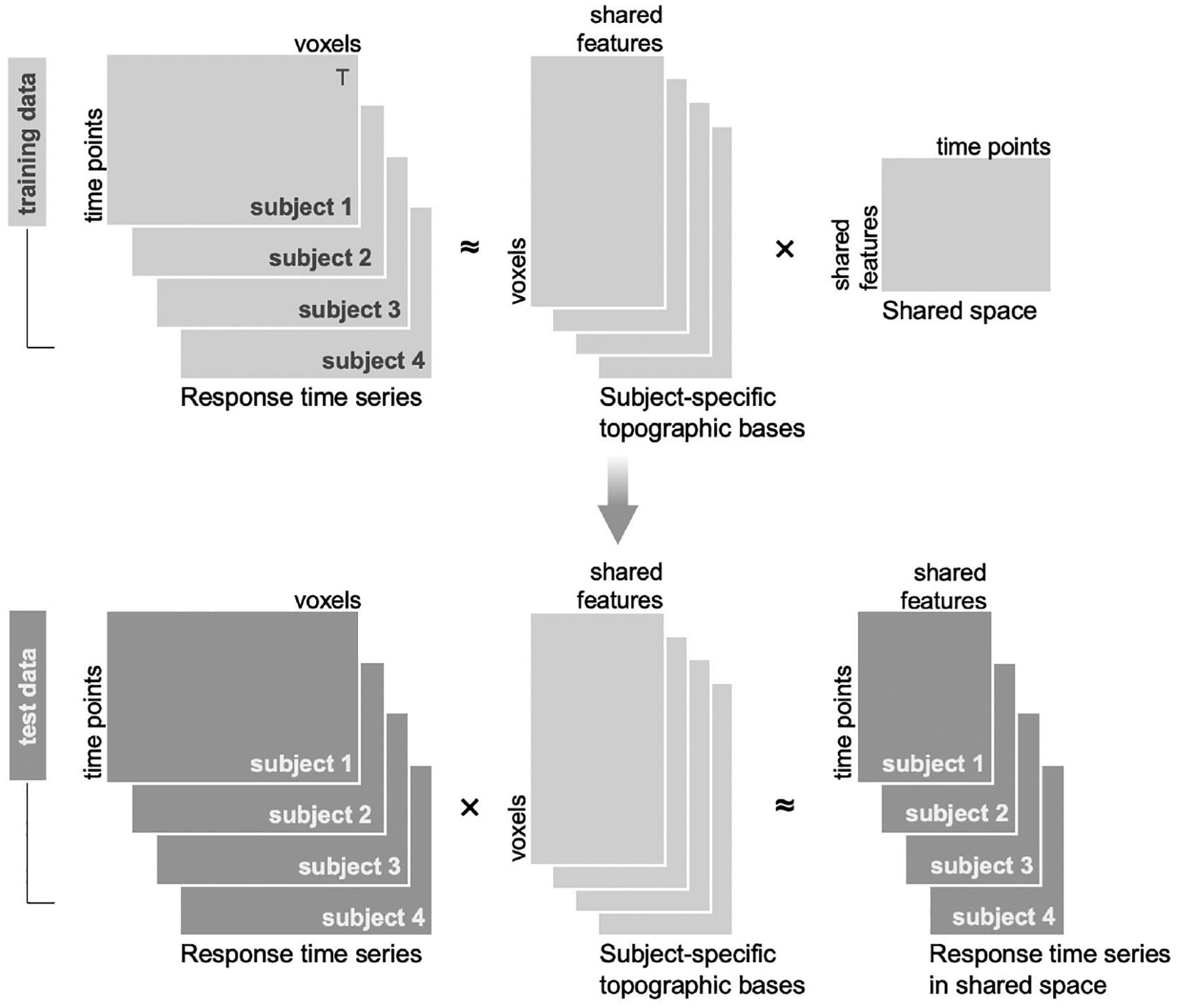
Schematic of the shared response model (SRM). Data are typically split into a training set (light gray) used to estimate the SRM and a test set (dark gray) used for evaluation. The SRM is estimated from response time series from the training set for multiple subjects (top left; transposed here for visualization). The multisubject response time series are decomposed into a set of subject-specific orthogonal topographic transformation matrices and a reduced-dimension shared response space. The learned subject-specific topographic bases can be used to project test data (bottom left) into the shared space. This projection functionally aligns the test data.

SRM [13], alongside other methods of hyperalignment [45–47], aims to resolve this alignment problem by aligning on the basis of functional data. SRM estimation is driven by the commonality in functional responses induced by a shared stimulus (e.g., watching a movie). Unlike ISC analysis, which presupposes (often very coarse) functional correspondence, SRM isolates the shared response while accommodating misalignment across subjects. SRM decomposes multisubject fMRI data into a lower-dimensional shared space and subject-specific transformation matrices for projecting from each subject’s idiosyncratic voxel space into the shared space (Figure 2). Each of these topographic transformations effectively rotates and reduces each subject’s voxel space to find a subspace of shared features where the multivariate trajectory of responses to the stimulus is best aligned. These shared features do not correspond to individual voxels; rather, they are distributed across the full voxel space of each subject; each shared feature can be understood as a weighted sum of many voxels.

Transformations estimated from one subset of data can be used to project unseen data into the shared space. Projecting data into shared space increases both temporal and spatial ISC (by design), and in many cases improves between-subject model performance to the level of within-subject performance. Between-subject models with SRM can, in some cases, exceed the performance of within-subject models because (a) the reduced-dimension shared space can highlight stimulus-related variance by filtering out noisy or non-stimulus-related features, and (b) the between-subject model can effectively leverage a larger volume of data after functional alignment than is available for any single subject (e.g., [13, 48]). Denoised individual-subject data can be reconstructed by projecting data from the reduced-dimension shared space back into any given subject’s brain space. Furthermore, in cases where each subject’s unique response is of more interest than the shared signal, SRM can be used to factor out the shared component, thereby isolating the idiosyncratic response for each subject [13].

Building on the initial probabilistic SRM formulation [13, 49], several variants of SRM have been developed to address related challenges. For example, a fast SRM implementation has been introduced for rapidly analyzing large datasets with reduced memory demands [50]. The robust SRM algorithm tolerates subject-specific outlying response elements [51], and the semisupervised SRM capitalizes on categorical stimulus labels when available [52]. Finally, estimating the SRM from FC data rather than response time series circumvents the need for a single-shared stimulus across subjects; connectivity SRM allows us to derive a single-shared response space across different stimuli with a shared connectivity profile [48].

#### The Notebook

The accompanying notebook applies the SRM to an example fMRI story-listening dataset from the “Narratives” data collection [39]. We apply the SRM within a temporal–parietal region of interest (ROI) comprising the auditory association cortex from a functional cortical parcellation [41] and explore the components of the resulting model. We evaluate the SRM using between-subject time-segment classification. This analysis reveals that the SRM yields a considerable improvement in between-subject classification beyond anatomical alignment.

#### Compute Recommendations

The computational demands to estimate the SRM scale with the number of subjects, duration of the data (number of TRs), the number of voxels in a given ROI, and the number of features requested. However, for a typical dataset comprising 30 subjects with 500 TRs worth of data, and an ROI containing 1,000 voxels, the SRM can be estimated on a personal computer in a matter of seconds. For large datasets (e.g., containing hundreds of subjects), we recommend using a parallel computing cluster. SRM estimation can be parallelized across ROIs.

### Full Correlation Matrix Analysis

#### The Problem: Computationally Tractable Analysis of the Complete Functional Connectivity Matrix

FC refers to coupling of activity in different regions of the brain; it is typically measured as the temporal correlation of BOLD activity across voxels. To assess FC in an unbiased way over the entire brain would require calculating the correlation across all pairs of voxels. However, given the number of voxels in most datasets, this is computationally challenging and results in data with very high dimensionality that are hard to analyze or interpret [53]. To address this, traditional analyses of FC have restricted the number of voxel correlations, either by using one or a small number of “seed” regions (preselected sets of voxels with which all others are correlated; akin to ROIs in standard analyses) or by parcellating the brain into larger regions [54] and then correlating the mean activities of the parcels. However, both approaches require assumptions and provide a coarse view of FC. Seed-based approaches are constrained to measuring FC with respect to only the seeds, and thus the selection of the seeds can bias the results. Parcel-based approaches are constrained by how the parcels are defined and assess FC with lower spatial resolution because multiple voxels are averaged per parcel.

#### The Solution

**Fig. 3.**
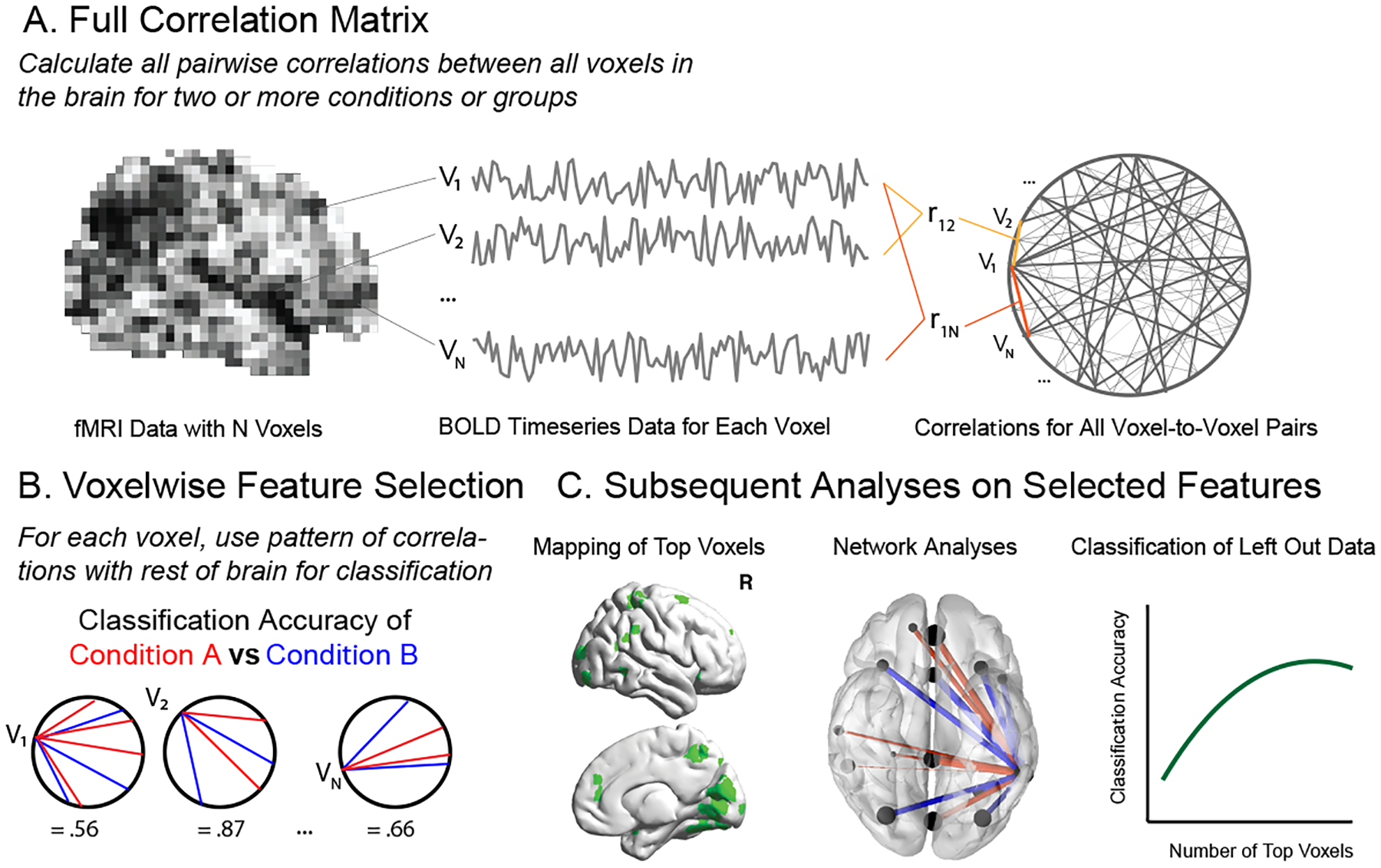
Full Correlation Matrix Analysis (FCMA). A. FCMA leverages several computing optimizations to permit calculation of full functional connectivity between all voxels in the brain. B. By default, FCMA then performs SVM classification on each voxel’s pattern of connectivity with the rest of the brain in order to assess how well each pattern differentiates two conditions or groups. C. The best performing voxels from B can then be used to guide additional analyses including visualizing/mapping top voxels, analysis of nodes and edges using graph theory-based metrics, and classification of patterns of connectivity from held-out data.

FCMA (Figure 3) is entirely data-driven and does not require the specification of initial seed regions or parcellations to reduce computational burden [55]. Rather, FCMA performs classification on the pattern of whole-brain connectivity for every voxel in the brain, effectively running all possible (usually thousands of) seed-based classification analyses at once. This provides a voxel-level measure of classification performance that can be used in several ways. First, this can serve as a form of feature selection, restricting further analysis of (independent) data to voxels with the best correlation-based classification performance. Second, it can drive discovery by revealing not only functional regions known to be involved in a task because of their activation but also regions previously overlooked because their FC but not activation is selective (e.g., [56]). That is, FCMA can reveal regions that are functionally coupled in a task-dependent manner without the use of a priori seed regions or parcellations, where these regions might not otherwise be found using standard activation-based analyses.

FCMA calculates the full correlation matrix at the voxel level, that is, the correlation of every voxel with every other voxel for any given set of time windows in a dataset. In multicondition, multisubject datasets, this is a massive computation: for example, a typical dataset with ~30,000 voxels has ~450,000,000 voxel pairs. The computational load only grows if the correlation matrix is computed for multiple time windows, as is often the case. To make this more tractable, FCMA leverages several optimizations, including high-performance kernels to calculate and classify correlations and Message Passing Interface (MPI) [57] to distribute the parallelizable tasks among multiple compute nodes. These optimizations make it possible to use the full correlation matrix computation in offline analysis and also in circumstances that require rapid calculation of FC data (e.g., real-time imaging, bootstrap hypothesis testing).

While it uses sophisticated algorithms to calculate the full correlation matrix, FCMA is intended to be accessible and highly flexible. FCMA relies on a customized, high-performance SVM classifier [58] and can be ported to other classification algorithms with scikit-learn-like interfaces. It can handle many different experimental designs and classification preferences (e.g., within- or across-subject classification).

#### The Notebook

The notebook illustrates the utility of FCMA across three steps. First, using a nested cross-validation procedure, it shows how to identify the set of voxels whose pattern of FC differentiates two hypothetical experimental conditions. Second, the notebook shows how to use FC in these selected voxels to successfully perform classification on held-out data. Third, it highlights how FCMA can provide useful results that can be visualized to test specific hypotheses or perform exploratory analyses.

#### Compute Recommendations

The computational demands of FCMA scale with the number of voxels and the number of epochs in the experiment. The calculation of all pairwise correlations across voxels results in a large memory footprint. For example, in a dataset with 30,000 voxels, one full correlation matrix with single precision number will take about 3.35 GB, and the analyses often require computation of multiple correlation matrices. We strongly recommend using either a workstation with a large amount of RAM or (ideally) a compute cluster to run FCMA.

### (Group) Bayesian RSA

#### The Problem: Unbiased Estimation of Neural Similarity Structure

RSA [2, 32, 59] is a method for quantifying the structure of the representational space in a brain region, either for external stimuli or for cognitive processes of interest. The traditional approach to RSA first estimates neural activity patterns for each task condition from fMRI data using the general linear model or directly uses raw fMRI patterns, and then calculates their pairwise (dis)similarity using metrics such as Euclidean distance, Mahalanobis distance, or Pearson correlation between the estimated patterns. As shown in several papers [14, 15, 60, 61], this approach can introduce a spurious similarity structure if neural patterns are estimated based on events happening close in time. This spurious similarity structure arises from the interaction between the autocorrelation in the task-unrelated fMRI fluctuations and the intrinsic correlational structure of the design matrices used when estimating neural patterns [14, 15].

#### The Solution

**Fig. 4.**
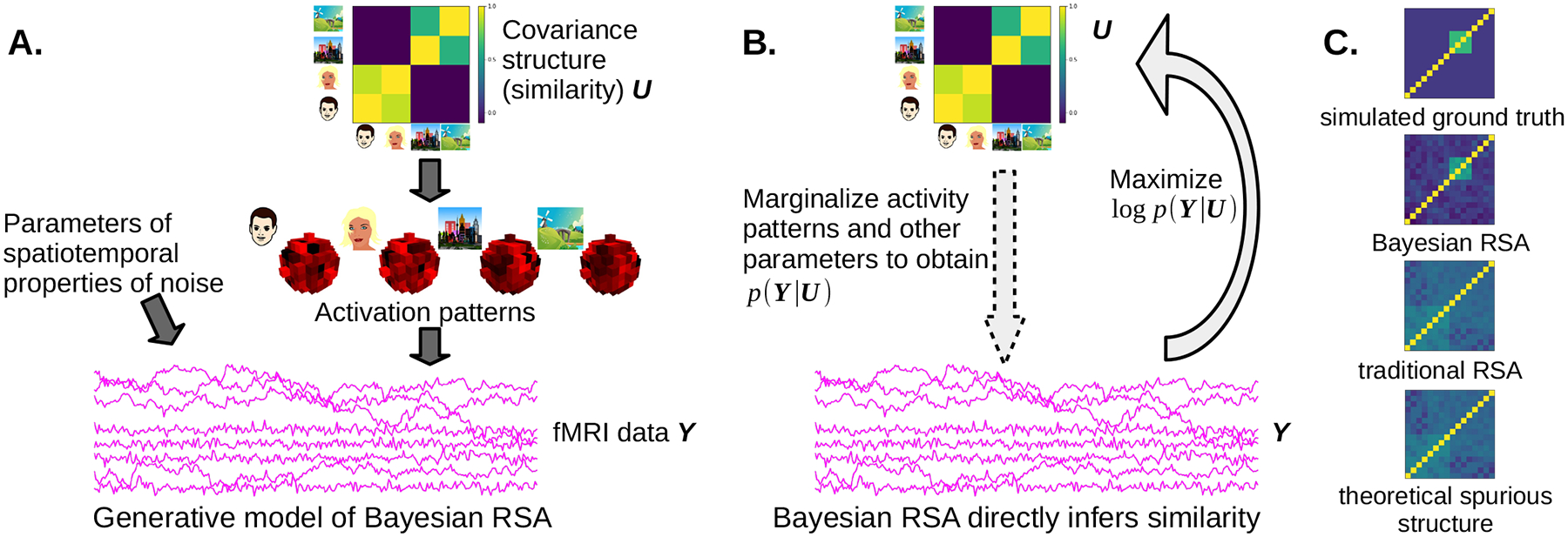
Overview of (G)BRSA. A. (G)BRSA assumes a hierarchical generative model for fMRI data, where a hypothetical covariance structure governs the distribution of response amplitudes of each voxel to different task conditions (here we take four images as an example), and the response amplitudes, in turn, contribute task-evoked responses to the fMRI data according to the design matrix. Other parameters determine the spatial and temporal properties of noise (and spontaneous activity). Arrows indicate causal relations in a probabilistic graphical model. B. (G)BRSA marginalizes out intermediate variables that contribute to fMRI data, making it possible to compute the log likelihood of the fMRI data Y given the covariance structure U (the arrow with dashed contour); the algorithm then finds U that maximizes this log likelihood (the solid arrow), which can be converted to a similarity matrix of activation patterns. C. BRSA significantly reduces bias (spurious similarity structure) compared to traditional RSA on a simulated dataset with 16 task conditions. The four figures are (from top to bottom) the ground truth similarity structure in the simulated data, similarity structure recovered by BRSA and traditional RSA, respectively, and the theoretically derived spurious structure arising from the interaction between fMRI noise and the design matrix (Figure C reproduced from [15]).

BRSA [14, 15] tackles this problem by simultaneously modeling two sources of contribution to the temporal correlation structure in the fMRI data: task-related signals and task-unrelated fluctuations. As shown in Figure 4A, it models the true task-related responses (defined as responses reproducible by a repetition of task condition) as samples drawn from a multivariate Gaussian distribution, the covariance structure of which underlies the representational similarity structure of interest. The spontaneous neural activity and scanner noise contribute additional spatial and temporal correlation to the data, which are explicitly modeled by BRSA. By marginalizing out the unknown spatial patterns of the neural response to each task condition, as well as the task-unrelated spontaneous activity patterns (Figure 4B), BRSA calculates the log likelihood of obtaining the whole-brain fMRI data given any possible covariance structure of the task-related response. It then searches for the covariance structure that maximizes the log likelihood, and converts this covariance structure to a correlational structure, which serves as an estimate of the representational similarity. This approach significantly reduces the confounding similarity structure arising from the interaction between task-unrelated fMRI signals and the deconvolution procedure for estimating neural activation patterns in traditional RSA (Figure 4C). In addition, BRSA can be extended to estimating representational structure from a group of participants, with the assumption that a common representational structure is shared by all participants. This approach is called Group Bayesian RSA (GBRSA; [15]). Notably, BRSA, like SRM (described earlier) and TFA (described here), is a low-dimensional factor model of fMRI data; these models only differ in their prior assumptions about the spatial or temporal properties of the factors and the quantities they aim to estimate [62].

#### The Notebook

BrainIAK’s reprsimul.brsa module contains two models: BRSA and GBRSA. The BRSA model follows the algorithm in [63], with the improvement that it also models spatial noise correlation. In addition to modeling spatial noise correlation, the GBRSA model also marginalizes voxel-wise parameters such as signal-to-noise ratio and temporal autocorrelation coefficients of noise and can estimate similarity structure from either a single participant or from a group of participants. The notebook accompanying this paper illustrates the usage of GBRSA on a group of simulated participants. Readers can easily adapt the example to the case of a single participant by providing only one participant’s data to the model. The notebook also illustrates additional functions of the model: decoding task-related signals from new data and cross-validating the fitted model to left-out data. It further provides tips for detecting false discoveries when the data contain too little task-related activity, with an example case of fitting a model to data composed of pure noise.

#### Compute Recommendations

The computational demands to estimate the BRSA scale with the cube of the number of task conditions. Fitting data on approximately 4,000 voxels, 720 TRs, and 16 task conditions on a 12-CPU Intel Xeon processor takes about 2,200 s. The computation time is spent mostly on inverting a covariance matrix during fitting and can be reduced by making simplifying assumptions as follows: (1) choosing “equal” for the option of SNR_prior by assuming all voxels have the same SNR; (2) reducing the parameter of rho_bins (e.g., to 10) to marginalize the autoregressive coefficient of the noise on a coarse grid; (3) when fitting a dataset with many task conditions, choosing a rank parameter smaller than the number of task conditions, which assumes the similarity matrix is a low-rank matrix. As low-level implementations of NumPy can automatically utilize multiple CPUs on the same computer, we recommend using multicore workstations or compute clusters for this analysis.

### Event Segmentation

#### The Problem: Tracking How the Brain Segments Continuous Inputs into Discrete Chunks

Foundational work in cognitive psychology [64, 65] has demonstrated that humans segment continuous inputs into discrete chunks (events). One way to study the neural basis of this chunking process is to have human annotators mark event boundaries [66] and then relate these human annotations to neural data. However, annotations are not always available, and other levels of chunking may be present in the brain besides the level corresponding to the annotations; as such, it would be beneficial to have a more data-driven way of studying how the brain chunks its inputs, other than relating on annotations.

#### The Solution

To address this problem, Baldassano et al. [16] introduced a hidden Markov model (HMM) approach designed to identify stable neural states at varying timescales. This model can be applied to responses during perception of one or more stimuli with aligned event structure [67, 68], to independent annotations or latent variables [69], or to align event structure between perception and free recall [16]. Although we describe its use for analyzing fMRI data, this model has also been used to analyze EEG data [70].

The HMM assumes that brain regions are always in some discrete (unobserved) event state. Our goal is to compute a probabilistic estimate of event identity at each timepoint (TR), given a TR × voxel array of the neural response to some stimulus. The model makes three key assumptions: (1) On every TR, the brain region stays in the same event as the last TR or advances to the next event; (2) the brain region starts in the first and ends in the last event; and (3) events are associated with distinct spatial patterns across voxels, such that the pattern at every TR within an event consists of this event-specific pattern plus random noise.

**Fig. 5.**
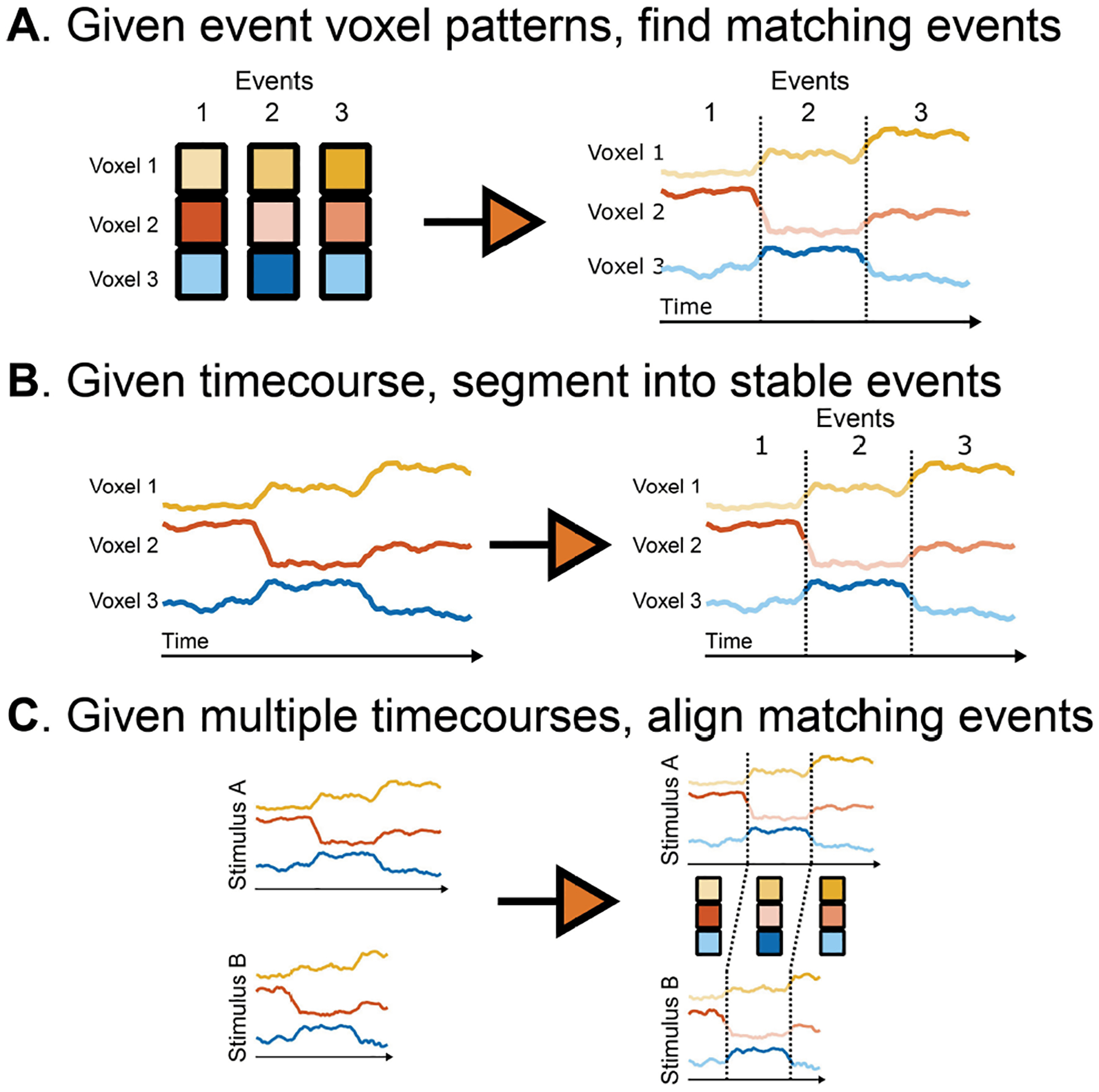
Use cases for the event segmentation model.

We can perform inference in the model in several ways, as shown in Figure 5:

- We can fit the model on a TR × voxel dataset by iteratively alternating between estimating event patterns and estimating TR probabilities for each event. The number of events must be prespecified, but cross-validation can be performed to determine the optimal number. This approach has previously elucidated how event structure can be represented at multiple timescales [16].
- If we already know the event-specific patterns (e.g., from an independent task), we can create a model with these patterns and infer event probabilities on a TR × voxel dataset of neural responses.
- If there are multiple datasets (with aligned voxels but different numbers of TRs) that share the same event sequence (e.g., responses to different versions of the same narrative, or to perception and recall of the same narrative), we can find shared event patterns across datasets and the per-TR event probabilities for each dataset.

#### The Notebook

The corresponding notebook demonstrates how to fit the HMM to real movie-watching data, align neural event boundaries with annotations, and apply the HMM to recall data [16]. Note that the eventseg package includes two extensions beyond the original paper [16]:

- You can define multiple “chains” of events rather than a single sequence. For example, if subjects recalled one of multiple stories, a separate event chain could be defined for each story and the model will assume that recall is equally likely within any separate chain.
- You can perform a more exhaustive fitting procedure when estimating the event patterns. This (slower) approach attempts to split events or merge neighboring events for better allocation throughout the time series.

It is also possible to run the HMM on other feature spaces rather than voxel activities. For example, the HMM can be run on the shared feature space constructed by SRM [13] (as in [68]) or on dynamic FC measures.

#### Compute Recommendations

The computational demands to fit the HMM model scale with the number of voxels, the number of timepoints, and the number of events. On a typical fMRI ROI (approximately 100s of voxels, 100s of timepoints, 10s of events), event segmentation can be performed in several seconds. Running with prespecified event patterns is about 10× faster, while using split-merge fitting is about 10× slower. In order to perform whole-brain searchlight analyses and/or permutation analyses, we recommend running model fits in parallel using a compute server.

### Topographic Factor Analysis

#### The Problem: Efficiently Describing Network Structure

As neural datasets are often large, studying network patterns that require huge (O(n^2^)) time and space to compute can be intractable (e.g., for discussion see [71]). One way to address this is the approach used by FCMA (i.e., using optimized computations to obtain the entire voxel × voxel correlation matrix; [56]). However, these full connectivity patterns (because of their size) can be challenging to work with in downstream analyses. Further, summarizing patterns of correlations often requires additional analyses whereby voxels are thresholded and/or grouped into spatially contiguous clusters or ROIs [72].

#### The Solution

**Fig. 6.**
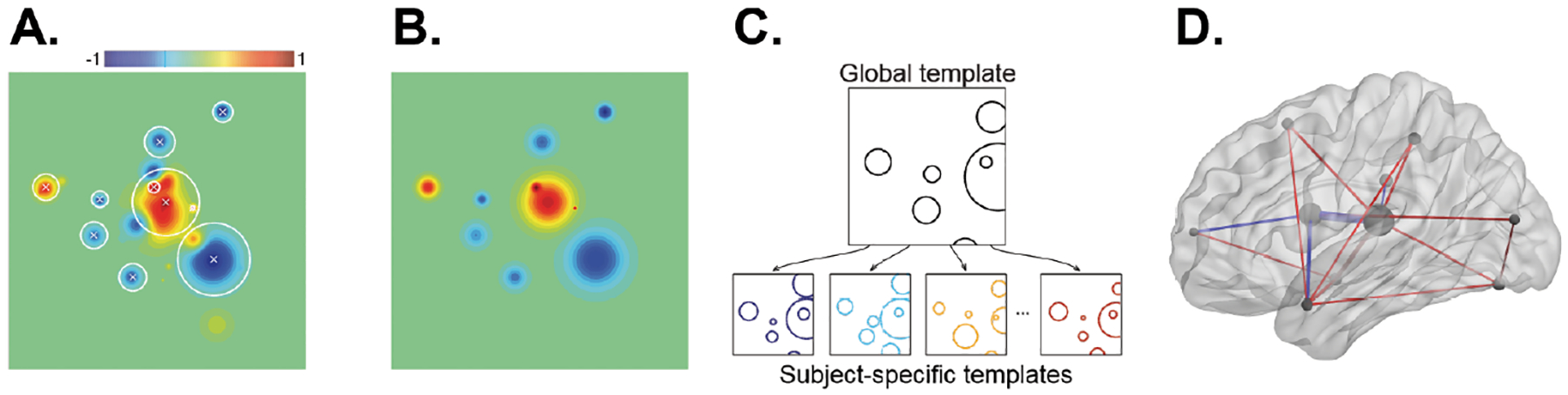
Topographic factor analysis. A. Spherical nodes describe contiguous sets of similarly behaving voxels. Each node is represented as a radial basis function. A node’s image may be constructed by evaluating its radial basis function at the locations of each voxel. Level curves for several example nodes fit to a synthetic 3D image are outlined in white; ×s denote the node centers projected onto the 2D slice displayed in the panel. B. Brain images are described by weighted sums of the nodes’ images. After computing each node’s image (using its radial basis function), arbitrary brain images may be approximated using weighted combinations of the images for each node. The per-image weights may be used as a low-dimensional embedding of the original data. A 2D slice of the reconstruction for the image displayed in panel A demonstrates how contiguous clusters of voxels are approximated using weighted activations of spherical nodes. C. The global template serves as a prior for subject-specific parameters. The global template defines the numbers of nodes, their locations, and their sizes, for the prototypical participant. Each individual participant’s parameters (node locations and sizes) are fit using the global template as a prior. This provides a linking function between different participants’ nodes, thereby enabling across-subject comparisons. A subset of the nodes outlined in panel A is displayed in the global template cartoon. The positions of these nodes in each individual participant’s subject-specific template are displayed in different colors. D. A “ball and stick” representation of network connections. The level curve of each node defines a spherical ball (gray). The per-image node weights may be used to infer static or dynamic functional connectivity patterns (i.e., correlations) between nodes: red “sticks” represent positive connections, blue sticks represent negative connections, and stick thickness is proportional to connection strength.

TFA [71, 73] takes a different approach, exploiting the strong spatial correlations in fMRI data (e.g., [74]) to derive a lower-dimensional description of the data that lends itself to efficiently characterizing full-brain connectivity patterns. Given a time series of 3D fMRI volumes, TFA finds a basis set of spherical “nodes” placed throughout the brain; each of these nodes represents a contiguous set of voxels (Figure 6A). (Nonspherical regions may be approximated using multiple spherical nodes.) Each brain image may then be described as a weighted sum of the images for each node (Figure 6B). When multisubject data are available, the locations and sizes of the nodes are constrained to be similar across people (Figure 6C). Applying TFA to a multisubject fMRI dataset yields a “ball and stick” representation of its underlying network dynamics (Figure 6D).

TFA works by defining a generative model for fMRI data. According to the model, data are generated by first choosing an appropriate number of nodes, K, and then assigning their locations and sizes within a global template that parameterizes a model of the “prototypical” participant (Figure 6C). Next, each individual participant’s nodes are selected by adding noise to the global template’s nodes (Figure 6C). In this way, this global template serves as a prior for the per-participant models, thereby ensuring that different participants’ nodes share similar locations and sizes. Finally, TFA assigns per-time-point activations to each node (Figure 6B). The fMRI volumes are generated by sampling the node activation patterns at the voxel sampling resolution of the images (Figure 6A). Applying TFA to an fMRI dataset entails “reversing” this generative process: given the fMRI data from each participant, the goal is to discover the most probable number of nodes, as well as the node locations and sizes, for each individual participant. TFA also estimates the global template, which may be used to summarize or align multisubject data (analogous to spatially warping fMRI data to align with a reference image).

By merging spatially nearby clusters of voxels whose responses are similar, TFA provides a highly efficient representation of neural data. Whereas approaches such as BrainIAK’s FCMA compute full-resolution brain correlation matrices at the level of individual pairs of voxels, TFA computes a lower-resolution approximation of full-brain correlation matrices (where the resolution depends on the choice of the number of nodes, K; the approximation becomes exact as K approaches the number of voxels). In this way, TFA is a convenient way of studying coarse spatial-scale full-brain network dynamics. A second useful property of TFA is its resolution independence. Because TFA’s nodes exist in “real space” rather than in the measurement space of the brain data (i.e., as voxels), the approach provides an elegant means of comparing or combining data at different resolutions.

#### The Notebook

In our companion notebook, we provide an example of how TFA may be applied to a multisubject fMRI dataset in order to examine the underlying network dynamics. We also provide several examples of how to visualize those dynamics using a variety of animations.

#### Compute Recommendations

The computational demands to apply TFA to a multisubject fMRI dataset scale primarily with the number of subjects (S) and the number of nodes (K). When run on a distributed computing cluster, the per-subject models may be fit in parallel. Fitting the model to a small fMRI dataset (S = 3 – 5 participants) using K = 10 nodes may be run on a modern laptop computer in roughly 20 minutes. A larger fMRI dataset (S > 50) and/or a large number of nodes K > 50 may be run overnight on a modern laptop computer. We recommend fitting large datasets with many subjects, or models with many nodes, on a distributed computing cluster.

### Inverted Encoding Model

#### The Problem: Incorporating Hypotheses about Stimulus Encoding into Decoding Models

Neural decoding algorithms estimate some function g(R) to map a measured neural response R to a stimulus S [1, 19]. Most of these decoding approaches are agnostic about how the stimuli are encoded in the brain (e.g., the use of a linear classifier like logistic regression simply assumes that stimulus classes are linearly separable in the space defined by the voxel activity patterns, without making any further assumptions about the mapping between stimulus properties and voxel activity values). This “encoding agnostic” approach may be appropriate in situations where little is known about how the stimuli are encoded [19]. However, in situations where researchers have clear hypotheses about how stimuli are encoded, building this information into the decoder could serve as useful source of constraints on the analysis, as well as a means of arbitrating between these hypotheses.

#### The Solution

**Fig. 7.**
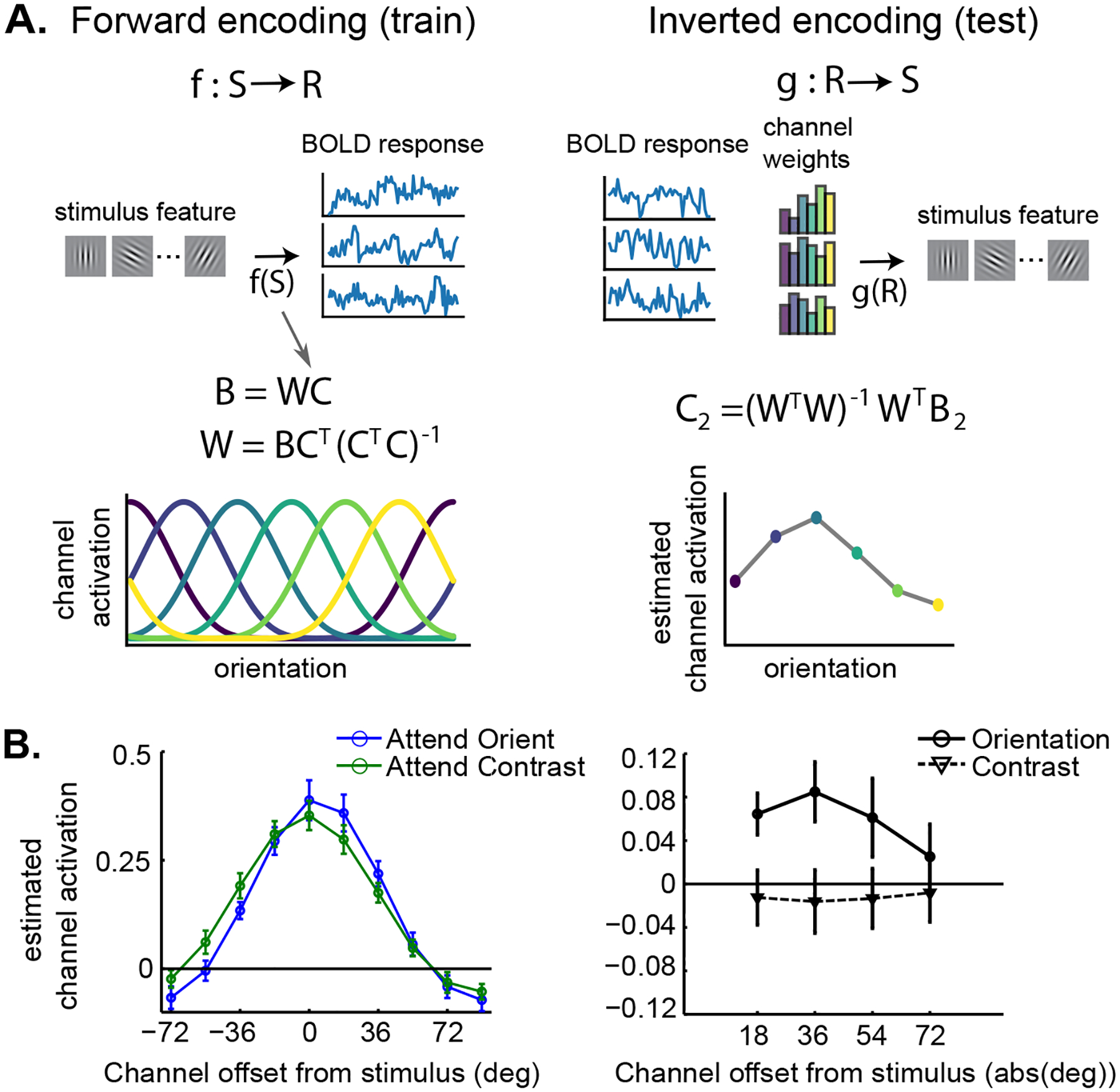
Inverted encoding model. A. Inverting the forward encoding model to reconstruct the stimulus feature, orientation. First, the experimenter specifies some nonlinear transformation of the stimulus into a representational space. Here, orientations of Gabor gratings are transformed into activations on a set of orientation channels C that tile the stimulus space. Then, the fMRI responses B are predicted by solving the linear equation B = WC. To reconstruct stimulus features with a new set of data B_2_, we simply invert W to predict a new C_2_. B. IEMs allow experimenters to test detailed hypotheses about stimulus representations. Scolari et al. [77] tested the off-channel gain hypothesis (figure adapted with permission). According to this hypothesis, when discriminating between very similar features, it is optimal to enhance the responses of channels close to the relevant feature, rather than directly enhancing the relevant feature. Using an IEM for stimulus orientation, Scolari et al. [77] demonstrated off-channel gain enhancement when subjects performed a difficult orientation discrimination task, compared to when subjects performed a contrast discrimination task on the same stimuli.

IEMs are designed to solve exactly this problem (i.e., of using hypotheses about encoding to inform how decoding takes place). The IEM approach involves first training an encoding model, which involves estimating some function f(S) to map stimulus features S to response R [18, 19]. Most encoding models assume that each voxel’s activity is determined by a weighted linear combination of a set of stimulus features (Figure 7). For example, Brouwer and Heeger [75] constructed features that tiled color space, assuming that each voxel had some distribution of sensitivity to these color features and solved for the weights W on those features. One can then define g(R) by inverting W to reconstruct the stimulus, yielding the IEM (Figure 7A). This approach makes it possible to predict output stimulus features never seen in the training set (e.g., predicting an orientation of 142° when only 120° and 150° were shown). As noted earlier, by incorporating assumptions that more closely match the structure of the data, the IEM can, in principle, be more powerful than other decoding approaches – that is, an IEM may be able to succeed in situations where linear decoders like Support Vector Machines (SVMs) fail [76].

The IEM also allows experimenters to address more nuanced hypotheses about stimulus encoding. For example, Scolari et al. [77] used it to test how attention shaped neural responses to oriented gratings under different conditions (Figure 7B). The reconstructed stimuli can also serve as a proxy for the representation in some ROI, for example, allowing experimenters to examine how the contents of visual working memory can be simultaneously represented with distracting perceptual inputs [78, 79]. Others have used the IEM to answer questions about prediction in the hippocampus [80] and memory-guided navigation in several regions of the brain [81]. See [82] for advice on the proper use of IEMs; further guidance is provided in the BrainIAK examples.

#### The Notebook

In the IEM notebook, we provide easily visualized reconstructions of one-dimensional and two-dimensional stimuli. Even with data from a single subject, we can begin to see how the experimental manipulations affect the stimulus reconstructions. We also provide simulations showing that SVM decoding results can be less accurate than IEM decoding results with small amounts of data.

#### Compute Recommendations

The computational demands to estimate the IEM scale with the number of voxels in a given ROI. Running this analysis (including training and testing the model) on a single subject and ROI takes a few seconds on a typical multicore laptop or desktop machine, as it relies on optimized matrix multiplication and singular-value decomposition operations. For large datasets, the analysis can be parallelized across subjects and regions either on a distributed computing cluster or a multicore machine with sufficient memory.

### fmrisim

#### The Problem: Simulating Realistic fMRI Data

Methods for analyzing fMRI data have blossomed in recent years, yet there is a concurrent need to understand how best to use these methods. Simulations of fMRI data can aid in both the evaluation of complex designs and the analysis of data. Software packages have been created that offer flexible simulation of fMRI data [83–85]; however, no package was designed explicitly for simulating data for multivariate analyses. Moreover, no available packages can generate simulated data with noise properties that are matched to an existing fMRI dataset.

#### The Solution

**Fig. 8.**
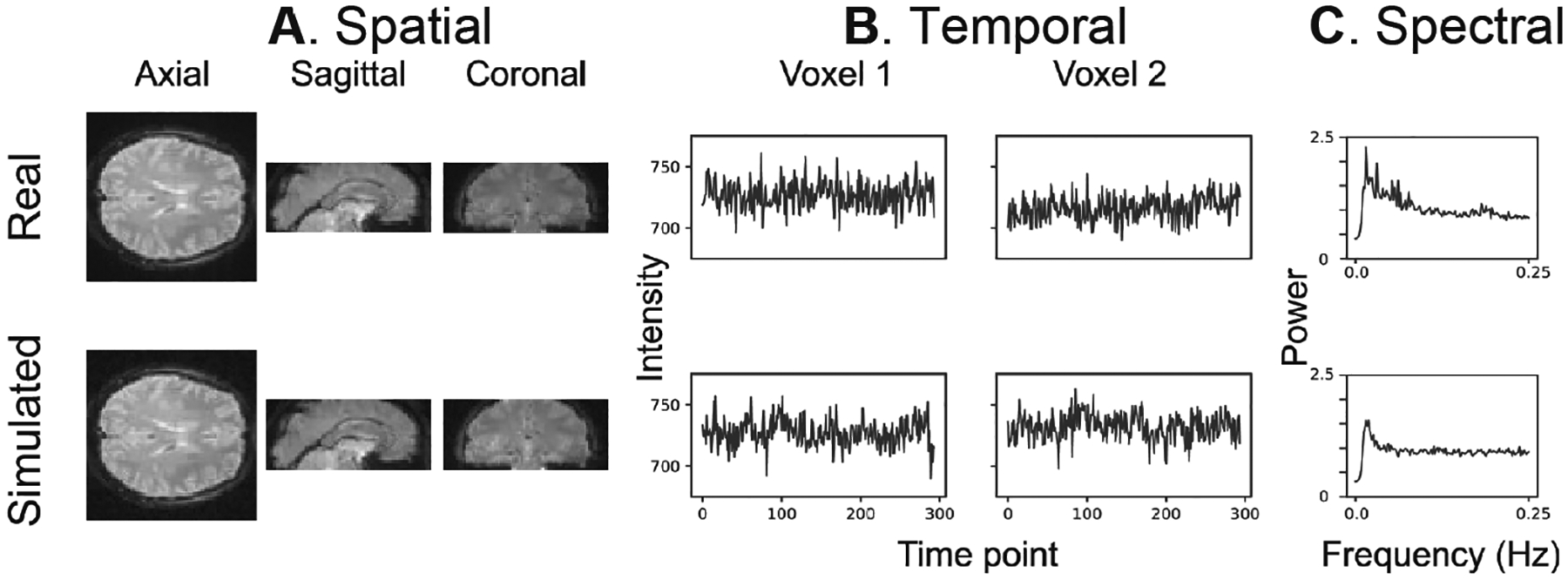
Example of the spatial and temporal structure of real and simulated data. The real data (top row) was input into fmrisim and produced simulated data (bottom row). A. It depicts the spatial structure of real data (top) and fitted simulated data (bottom). B. It shows the time course of sample voxels, and C. it shows the power spectra of a sample of high-pass filtered voxels. Reproduced from [23].

To fill in this gap, we developed fmrisim [23], an open-source Python package for simulating realistic fMRI data. fmrisim linearly combines a number of noise sources, inspired by biology and MRI physics, which are tuned in a data-driven fashion to match specific fMRI data that is provided as an input. Through an iterative fitting procedure, the noise properties of the simulation are updated to optimize the match of the simulated data to the real data (Figure 8). We previously validated that this fitting procedure produces accurate simulations of real data [23]. We have used fmrisim to evaluate the power of different experimental design parameters [23] and also to evaluate the efficacy of new analysis methods [86, 87].

fmrisim can be utilized in two main ways by researchers. First, it can be used to explore and optimize different experimental design parameters and analysis pipelines. This is particularly valuable in the case of complex, multivariate designs where traditional methods for evaluating design efficiency [88] may be inappropriate. Second, fmrisim can be used to preregister an experiment design and analysis pipeline to conduct confirmatory hypothesis testing. By establishing an analysis pipeline before any data is collected, simulation can be used as a sandbox to tune the analysis pipeline for testing a specific hypothesis, without compromising any real data. Hence, fmrisim offers a unique opportunity to conduct explicitly confirmatory research with fMRI. Considering these use cases together, we believe fmrisim is a valuable tool to help researchers conduct more reproducible fMRI research.

#### The Notebook

The corresponding notebook for fmrisim illustrates the simulation of a dataset and how it can be used for analysis. The hope is that this can be used as a template for simulating your own study. This notebook takes in an example functional dataset and simulates new data with the same noise properties as this real data. It performs each step of noise simulation individually, in order to give the reader a sense of what is being done. Signal is then inserted into the data. The signal is a multivariate pattern of voxel activity evoked by events from different fictitious conditions. Classification analysis is then performed to evaluate these block differences.

#### Compute Recommendations

The computational demands to use fmrisim to generate datasets are not large; fmrisim generates average-sized datasets in reasonable time frames on a single computer core (e.g., a personal laptop). For instance, to simulate a realistic run (294 TRs) of data in a total of 17 participants, it takes 4,371.1 s (278.3 s per participant) [23]. Generation of runs and participants can be parallelized on a cluster to make it trivial to simulate a full dataset in less than 5 minutes.

### Emerging Methods

This section of the paper describes new tools that are coming soon to the BrainIAK toolbox or were just added.

#### Topological Data Analysis and Geometrical Analysis

Innovations in TDA have generated remarkable new insights in neural coding [89–91]. The BrainIAK Extras repository provides a wrapper for PHAT [92], a C++ library for high-performance persistent homology. Future extensions may include wrappers for Rivet [93], a C++ package for multiparameter persistent homology.

Alongside TDA, geometric methods are starting to gain traction in neuroscience data analysis [91, 94], specifically as a tool to study how entangled and disentangled feature dimensions interact to determine neural codes across multiple contexts [95, 96]. Lightweight implementations of these methods are currently being developed in BrainIAK for investigating context-dependent cognitive feature representations. These implementations will include several practically motivated techniques to address problems associated with large and/or incomplete datasets, as well as diagnostic tools for cross-validation of findings. Utilities for efficient organization and formatting of user data will also be included.

#### Matrix-Normal Models

Many models for fMRI analysis are framed as linear regression or factor models with Gaussian noise. This includes variants of SRM, RSA, TFA, and ISFC (all discussed earlier in this article), the conventional fMRI generalized linear model (GLM), and others. Typically these models assume independently normally-distributed residuals in either the spatial or temporal dimension (and often both). To match the data to these independence assumptions, traditional approaches often preprocess their data to remove spatiotemporal correlations altogether. However, if the preprocessing model is misspecified (which is always true to some extent), fully removing these correlations removes some signal alongside the noise. In contrast, structured-residual models, herein called matrix-normal (MN) models, choose to jointly model the “signal” alongside the “noise” or residual covariance, letting the same model apportion signal relative to noise. Shvartsman et al. [24] proposed to introduce spatiotemporally structured-residual covariance to a number of the models discussed earlier, showing improved reconstruction performance for MN-SRM and faster and more conservative behavior for MN-RSA, as well as a derivation of matrix-normal ISFC (which is shown to be highly similar to SRM, mathematically). To enable further prototyping of fMRI models with spatiotemporally structured residuals, BrainIAK includes a model prototyping toolkit for such models, as well as examples of matrix-normal variants of some existing methods.

## FUTURE DIRECTIONS: REAL-TIME FMRI ANALYSIS

Real-time (RT) fMRI is an emerging technology that can be used to provide cognitive training to participants inside of the MRI scanner [97, 98]. Participants can receive neurofeedback (i.e., information about their current neural state) to help them modify their thinking to achieve a certain goal (e.g., increasing the amount of activation within a brain ROI, given a thermometer visualization as feedback). Researchers can also use an adaptive experimental design where they adjust stimuli in response to the participant’s present neural state, with the goal of driving the system into a desired neural state. These two types of neurofeedback have been effectively used in numerous studies, both in nonclinical (e.g., [99–102]) and clinical populations (e.g., [20, 103–106]).

Supporting real-time analysis is a major goal of the BrainIAK project going forward. In this section, we describe our framework for incorporating real-time analysis into BrainIAK, and we provide an accompanying notebook that demonstrates this framework.

### The Problem: Making Real-Time fMRI Analysis More Accessible

Implementing a real-time experiment currently has significant barriers to entry. Computer processing during real-time runs – including detecting and loading MRI images, performing image registration, updating classification models, providing participant neurofeedback, and recording subject responses – must be completed quickly enough to provide timely feedback (ideally within 1–2 seconds). These processes generate high computer load and require network communication between computers in real time; successfully implementing this kind of pipeline requires diverse IT and programming skills. Existing software frameworks have helped researchers to better implement RT-fMRI studies (e.g., [107–111]). However, many of these packages have one or more issues that limit their usability; for example, they may require licensed software or advanced computing skills on the part of the researchers.

### The Solution

**Fig. 9.**
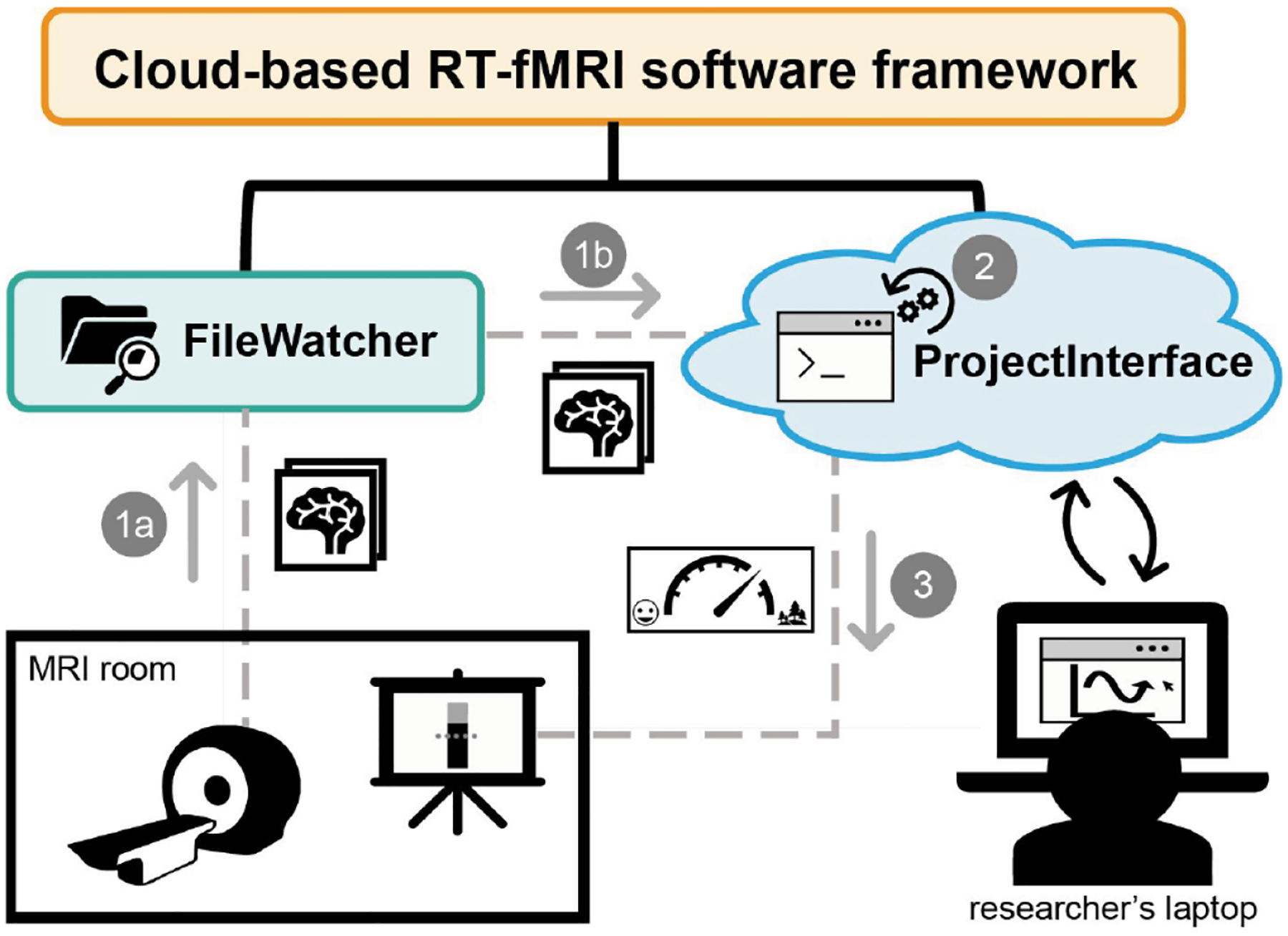
Schematic of our cloud-based software framework for real-time fMRI experiments. The framework has two main components: the FileWatcher and the ProjectInterface. (1a) The FileWatcher watches for the arrival of new DICOM images on the scanner computer and (1b) forwards the image to the ProjectInterface, running on the cloud. (2) The ProjectInterface, which wraps the experimenter’s code, processes the DICOM data and runs the experimenter’s analysis code to obtain a measure of the participant’s brain state. The experimenter accesses the cloud application from a browser page that can run on a laptop. Among other things, the experimenter can initiate/finalize the session, change settings, and even observe the graph output of the analysis results. (3) The analysis results are provided to the participant as visual neurofeedback presented on the projector in the MRI room.

Our goal is to make RT-fMRI more easily accessible to neuroscience researchers. To do this, we are developing a software framework, described in Figure 9, which streamlines the process of developing experiments, allowing the researcher to focus on only the code specific to their experiment. Our framework uses cloud computing, which mitigates the economic burden of buying hardware and makes experiment setup easier by eliminating the requirement to install hardware and software in the control room. It also uses a Software-as-a-Service (SaaS) model, which provides a consistent remote installation accessed through a web browser. With the SaaS model, users do not need to maintain their own software installations, thereby avoiding potential problems with OS versioning, library mismatches, and memory limitations. The SaaS model also allows for remote testing and configuration. Unlike a typical SaaS that uses a specific cloud service, our framework allows users to run RT-fMRI data analyses on their choice of system (including their institution’s own computing cluster), which can help users meet regulatory requirements. The combination of using cloud computing and the SaaS model in our framework has the potential to facilitate growth in the field – for example, by making it easier to deploy the system in hospitals and other clinical settings that might benefit from RT-fMRI research.

### The Notebook

Our companion notebook walks through an example of running our real-time software pipeline. It uses a sample script that builds and then applies a multivariate pattern classifier to synthetic fMRI data. The synthetic data is generated using the BrainIAK simulator (fmrisim) and is transferred to the sample script, in the Jupyter notebook, for processing. In a real deployment, this processing would be running in the cloud. Importantly, the companion notebook implements a simplified version of the framework; readers interested in using the framework for full-scale real-time studies should visit the main rt-cloud repo at https://github.com/brainiak/rt-cloud.

### Compute Recommendations

The computational demands to run a real-time analysis on the cloud or cluster virtual machine are dependent on the experimenter’s choice of registration and classification methods, as well as the scanning parameters (such as the TR interval). We do not recommend running real-time analysis on a personal computer.

## TUTORIALS

### The Problem: Learning Advanced fMRI Analysis

It is usually quite challenging for a new user to learn advanced fMRI analysis. There are three main challenges in applying BrainIAK methods. First, one needs to learn Python, a language that has only recently gained traction in psychology and neuroscience departments. Second, the analyses require knowledge of machine learning techniques that may be unfamiliar to cognitive neuroscientists. Third, these methods need to be executed on HPC clusters, a task that is nontrivial for even advanced practitioners.

### The Solution

To overcome the challenges of applying BrainIAK methods, we have created a set of tutorials for advanced fMRI analysis (https://brainiak.org/tutorials) that are user-friendly, free to use, and open source [22]. These tutorials complement other learning resources that have become widely available: for example, Nilearn documentation (https://nilearn.github.io/stable/auto_examples/index.html), scikit-learn tutorials (https://scikit-learn.org/stable/auto_examples/index.html), the Neurostars forum (https://neurostars.org) [112], and Neurohackademy videos (https://neurohackademy.org/course_type/lectures/).

The BrainIAK tutorials cover 13 topics in fMRI analysis: setup, data handling, classification [1], dimensionality reduction, classifier optimization, RSA [2, 32] searchlight [113], seed-based connectivity, FCMA [56], ISC [10] and ISFC [11], SRM [13], event segmentation [16], and real-time analysis [101]. Each tutorial is a Jupyter notebook [114] that provides a step-by-step introduction to one method. The materials are designed for individuals with only basic knowledge of fMRI, cognitive neuroscience, and Python coding. Hence, detailed background information is provided for each method and dataset to introduce novices to the material. We show users how to avoid pitfalls like circular inference [115], and handle complexities that arise when working with large datasets. Moreover, the code is commented to support learning. Exercises are provided to encourage a deeper understanding of the methods. The methods are integrated with Nilearn functions for loading and manipulating neuroimaging data and machine learning functions from scikit-learn. The tutorials are run on preprocessed datasets taken from experiments published in the literature. These datasets include block design experiments, naturalistic movies, people listening to stories, and simulated datasets for real-time analysis. To scaffold the transition to using a high-performance computer cluster in order to analyze data at scale, we also explain how to use a Slurm scheduler and how to estimate memory and execution time for jobs, and we provide batch scripts to help the learner acquire this complex skillset. These tutorials have now been used in semester-long courses at Princeton and Yale. The feedback from these courses, along with those from hackathons and workshops, has helped to refine the tutorials.

### The Notebooks

The tutorials are available at https://brainiak.org/tutorials. They are modular and can be run independently. We provide recommendations on where to start based on the skill level of the user.

Hackathons are a particularly suitable environment to utilize these tutorials since new participants can both learn from these tutorials and improve their documentation. To increase accessibility and flexibility for different usages, we provide a variety of installation options for the tutorials with a Docker container, Conda, and even a cloud option using Google Colaboratory.

## HPC: OPTIMIZATIONS AND SCALABILITY

Several of the methods in BrainIAK incorporate algebraic, algorithmic, and computing optimizations that make it possible to scale the analyses to high-resolution images and large numbers of subjects when run on HPC clusters. BrainIAK uses parallel and distributed processing via Python multiprocessing as well as standard HPC technology (OpenMP and MPI) and has been tested and used heavily on HPC clusters that use the Slurm scheduler. However, it is written to be agnostic to which HPC scheduler is used. With modules that utilize single-node multiprocessing, the default number of processes is configured to observe and respect Linux control groups (cgroups) cpuset restrictions. Many Linux-based HPC schedulers can use the cgroup subsystem to restrict available processing units for processes that have reserved only a portion of available cores on a node. This should prevent novice users from having under or over-provisioned default configurations of these algorithms on single-node shared HPC resources. For algorithms that implement multiple-node parallelism, MPI (using the mpi4py Python package) is used; we have tested this with both OpenMPI and MPI over Chameleon (MPICH) implementations, though others will likely work. The proper configuration of these libraries and running of these types of jobs under a scheduler is left up to the user to determine. We highlight here the optimizations in BrainIAK that enable the methods to run at scale.

Searchlight analysis is a good target for a scalable implementation, since all searchlights are independent and can hence be executed in parallel. Searchlight analysis performs a separate configurable analysis for every voxel in the brain by passing a moving window over the brain and limiting consideration to only local voxels at each step [113]. Our implementation automatically parallelizes over the cores available in the CPU using Python multiprocessing, even when running on a laptop. The same code can be run unmodified on an HPC cluster and can run in parallel over cluster nodes if launched as a set of MPI processes. Input data are automatically distributed in the cluster from the first MPI process and output is automatically collected. The data distribution across processors can be done by either distributing each subject to a different processor or splitting sets of voxels to different processors. Finally, to help nonexperts take advantage of cluster execution, we provide a comprehensive tutorial that includes scaling advice, for example, estimating memory requirements (see tutorial: https://brainiak.org/tutorials/07-searchlight/).

In FCMA [58], the most computationally-intensive part of the analysis involves a three-stage pipeline: correlation computation, within-subject normalization, and voxelwise SVM cross-validation. To optimize for modern processors, we reduce the computation of Pearson correlation between voxel pairs to the multiplication of a voxel-by-time matrix and its transpose, by normalizing the data within each time epoch. Although many libraries such as Intel MKL have been optimized extensively for matrix multiplications, they do not perform well for whole-brain fMRI datasets, which typically involve tall-skinny matrices with large numbers of voxels and few timepoints. We employ several special optimizations for modern processors. First, we partition tall-skinny matrices into block matrices to fit the small amount of memory built into the CPU (L2 cache) for each thread. Second, the cache contents are retained across stages of the procedure pipeline by merging (fusing) two computation stages. In other words, when the current stage finishes the computations of a blocked matrix, it proceeds with the next-stage computation of this block without waiting for other blocks of the current stage to complete. Third, we carefully design data structures and workflow for vectorization. Such optimized single-node code runs 1.5×–2.5× faster than using Intel MKL and LibSVM libraries on Intel Xeon processors and 5×–16× faster than that on Xeon-phi processors. The parallel implementation of FCMA for computer clusters achieves near-linear speedups.

In SRM [49], the initial formulation of the problem requires the inversion of a square matrix whose size is the number of voxels by the number of subjects. We use the matrix inversion lemma and other linear algebra transformations to require only memory proportional to the number of SRM features, much lower than the number of voxels. We support parallelizing the computation by subject, using MPI. Finally, we minimize the data sent between MPI processes, which may otherwise become a bottleneck when running on an HPC cluster. With the FastSRM algorithm [50], we can apply SRM to large datasets that do not fit in memory. Its efficient implementation (relying on an intermediate atlas-based representation) yields similar performance to the initial formulation while being faster and more memory efficient.

In hierarchical TFA [49], the main bottlenecks we dealt with were large memory requirements for storing a certain Jacobian matrix and a large number of matrix inversions computed by an unconstrained nonlinear least-squares solver. To address the memory issue, we partition the model variables that determine the matrix size into two blocks; this doubles the required computations, but we consider this tradeoff to be worthwhile. To reduce the number of inversions, we use a constrained solver and apply the matrix inversion lemma. Parallel processing within-subject is implemented with OpenMP and across subjects with MPI.

## CONTRIBUTING TO BRAINIAK

BrainIAK follows open collaboration principles. While the Princeton Neuroscience Institute and Intel Labs started the project, contributions are welcome from anyone. Contributions can take many forms: Python code for one of the analysis methods, C++ code for speeding up computation, Jupyter notebooks to showcase method usage, documentation, bug descriptions, or community interaction via email and chat. To encourage new contributors, we advertise simple tasks on our public issue tracker on GitHub.

Source code is published on GitHub under an open-source license (Apache 2.0, except for the brainiak extras package, which uses LGPL 3.0 because of its dependencies). The criteria for accepting pull requests are documented in the contributing guide on GitHub and our site. In general, we follow scikit-learn guidelines. The requirements are automatically verified using GitHub Actions and CodeCov and must be satisfied before we accept a pull request. To help contributors run the same tests on their machines while developing, we provide scripts that call tools like flake8 or pytest with the right configuration.

Documentation is essential for attracting users and contributors. Therefore, we require each code contribution to be accompanied by documentation using the NumPy format. Furthermore, we provide both simple examples and comprehensive tutorials for most of the methods. Contributions improving the documentation are welcome.

All pull requests must be accepted by at least one reviewer. We strive to find multiple reviewers with expertise in both the software engineering and neuroscience aspects of the contribution.

We have a public email list and a public chat room for community discussions. We try to provide a welcoming environment for anyone to discuss issues less formally than via GitHub.

We found hackathons to be an effective way to attract new contributors. In addition to BrainIAK-specific hackathons we organized, we also submitted BrainIAK topics for larger hackathons, such as the one organized by OHBM.

## SUMMARY

Our goal in writing this article is to present an overview of BrainIAK as it stands at the time of publication, highlighting the various analysis methods incorporated in the toolkit, key themes linking these methods (e.g., making them HPC-friendly), and also key future directions (e.g., real time). By focusing on the problem addressed by each analysis method and providing example notebooks, we hope to have given potential users a sense of why they might want to use each method and how that method works. However, the descriptions here are brief and do not cover the techniques in detail. For readers interested in learning more about these techniques, we encourage them to follow the links in the annotated bibliographies that accompany the notebooks and also (when applicable) to the relevant tutorials.
